# Are there right hemisphere contributions to visually-guided movement? Manipulating left hand reaction time advantages in dextrals

**DOI:** 10.3389/fpsyg.2015.01203

**Published:** 2015-08-28

**Authors:** David P. Carey, E. Grace Otto-de Haart, Gavin Buckingham, H. Chris Dijkerman, Eric L. Hargreaves, Melvyn A. Goodale

**Affiliations:** ^1^Perception, Action and Memory Research Group, School of Psychology, Bangor UniversityBangor, UK; ^2^School of Psychology, University of AberdeenAberdeen, UK; ^3^School of Life Sciences, Heriot-Watt UniversityEdinburgh, UK; ^4^Experimental Psychology, Helmholtz Institute, Utrecht UniversityUtrecht, Netherlands; ^5^Division of Neurosurgery, Robert Wood Johnson Medical School, Rutgers UniversityNew Brunswick, NJ, USA; ^6^Brain and Mind Institute, Western UniversityLondon, ON, Canada

**Keywords:** cerebral asymmetry, reaching, handedness, visuospatial processing, attention, reaction time

## Abstract

Many studies have argued for distinct but complementary contributions from each hemisphere in the control of movements to visual targets. Investigators have attempted to extend observations from patients with unilateral left- and right-hemisphere damage, to those using neurologically-intact participants, by assuming that each hand has privileged access to the contralateral hemisphere. Previous attempts to illustrate right hemispheric contributions to the control of aiming have focussed on increasing the spatial demands of an aiming task, to attenuate the typical right hand advantages, to try to enhance a *left hand* reaction time advantage in right-handed participants. These early attempts have not been successful. The present study circumnavigates some of the theoretical and methodological difficulties of some of the earlier experiments, by using three different tasks linked directly to specialized functions of the right hemisphere: bisecting, the gap effect, and visuospatial localization. None of these tasks were effective in reducing the magnitude of left hand reaction time advantages in right handers. Results are discussed in terms of alternatives to right hemispheric functional explanations of the effect, the one-dimensional nature of our target arrays, power and precision given the size of the left hand RT effect, and the utility of examining the proportions of participants who show these effects, rather than exclusive reliance on measures of central tendency and their associated null hypothesis significance tests.

## Introduction

The idea of a specialized role for the left hemisphere in the control of movement is well-established in the neuroscience literature (Kimura and Archibald, 1974; Paillard, 1982a,b; Goodale, 1988; Kimura, 1993; Elliott and Roy, 1996; Rothi and Heilman, 1997; Goldenberg, 2013). Nevertheless, surprisingly few investigations have examined the relative contributions of the two hemispheres to the programming and control of movement. One approach to this question has been to contrast differences in the movements made by groups of unilateral brain-damaged patients. To date, however, little consensus has been reached in these experiments, except for the general tendency for right-brain damaged (RBD) participants to initiate their movements more slowly than their left-brain damaged (LBD) counterparts (Fisk and Goodale, 1988; Haaland and Harrington, 1989, 1996). This result (and similar results from the hand difference literature using neurotypical participants, see below) is usually interpreted in terms of some sort of right-hemisphere process that is important for: (1) localizing a target in space; (2) shifting or allocating attentional resources; or (3) “premotor processing” [the latter tends mainly to refer to any processes related to the reaction time (RT) period].

Inferences derived from deficits following brain damage, on their own, can be difficult to interpret unambiguously (Kosslyn and Intrilligator, 1992; Shallice and Cooper, 2011). Hypotheses about hemispheric contributions to movement would be strengthened if they were supported by independent evidence from other research domains. One such domain is the study of hand differences in neurologically-intact participants. Given the “privileged access” of each hand's motor outflow and sensory inflow to other mechanisms in the contralateral hemisphere, subtle differences in the performance of the left and right hands should, in theory, be consistent with the specializations of each hemisphere (e.g., Goodale, 1988, 1990; Poizner et al., 1990; Bagesteiro and Sainburg, 2002). One result, commonly reported in the visually-guided aiming literature, is that left-handed movements are initiated more quickly (e.g., Carson et al., 1992; Carson, 1996), while right-handed movements are completed more quickly once initiated (e.g., Elliott et al., 1993, 1994).

Of course, the majority of hand performance studies have investigated right-handed participants and reported advantages for the right hand. The most robust of these advantages is **a** shorter dominant hand movement duration (Fisk and Goodale, 1985; Carson et al., 1993a,b; Elliott et al., 1994). Accuracy usually favors the right hand as well, suggesting that these shorter movement times (and higher peak velocities) are not an obvious result of a speed-accuracy trade off, at least for these measures of speed.

A potentially more promising approach than simply documenting any obtained hand difference, has been to manipulate task demands in some fashion and make one-tailed, directional predictions about shifts away from advantages for a specified hand in right-handed participants (Watson and Kimura, 1989; Carson et al., 1990, 1992; Elliott et al., 1993). Three such studies are reviewed in detail by Carson (1996). Effectively, most of these studies have manipulated some feature related to the target of an aiming movement, which was thought to increase the spatial demands of the task.

For example, Carson et al. (1992) had participants extrapolate from a spatio-temporal pattern of targets to determine a reach endpoint which completes the figure. Four such figures were used, which depicted linear, quadratic, cubic, and quartic functions. The assumption made by the authors was that the number of “reversals” in a pattern predicted the spatial complexity-linear the least spatially complex, quartic the most spatially complex. The authors did not find the expected differences as a function of target.

Other published attempts at increasing the spatial complexity of an aiming task which have been investigated include interpolating the center of circles of different sizes (Elliott et al., 1993, experiment 1), reaching quickly and accurately to one of two different types of targets in one of two different locations (Elliott et al., 1993, experiment 2); and pointing to the mirror image of a target's location (Chua et al., 1992). Although the stimuli used in such tasks are plausible as spatially complex in some respects, the tasks that the participants are required to complete may not be. For instance, in some of the tasks participants actually had to reach to an identical position for each of the different targets (Carson et al., 1992; Elliott et al., 1993). In Elliott et al. (1993), for example, the circles of different diameters were always centrally positioned on paper backgrounds which required identical movement amplitudes to point to their centers.

Specific methodological details aside, these approaches tend to make broad assumptions about what constitutes a spatial manipulation. Unfortunately to date they also tend not to work (in terms of increasing or attenuating left hand RT advantages). In fact, many of the null effects of task in these experiments led Carson (1996) to conclude that any right hemispheric contributions to left hand reaction time advantages “do not arise from an engagement in spatial co-ordinate processing” (p. 163). In other words, Carson argues that whatever mechanisms drive the left hand RT advantage, they don't seem to relate to visuospatial processes.

In the current study, we explored the hypothetical right hemispheric driver of the left hand RT advantage with three different experiments. Our main aim was to identify a manipulation which would affect the size of the left hand RT advantage, providing more direct evidence that this hand difference is a consequence of a right hemisphere process. For two of the tasks, we were motivated by independent evidence suggesting right hemispheric specialization, in experiment 1 (bisection) and experiment 3 (the gap effect). This latter study also constituted a more direct test of an attentional, rather than a strictly visuospatial, contribution to the left hand RT advantage, rarely done before (see Mieschke et al., 2001 for a noteworthy exception). For the remaining task, we attempted to manipulate spatial processing by altering the number of potential targets (experiment 2). In our first experiment, we attempted to circumvent the difficulties associated with defining spatial complexity using the old-fashioned approach of avoiding it altogether, by using a task with a known right hemispheric specialization (i.e., bisection of the space between two targets).

In all of the studies described below, participants were encouraged to make quick and accurate movements, but after early testing in one of our labs, we elected to emphasize speed more than accuracy in subsequent experiments where non-dominant hand performance was assessed alongside dominant hand performance. Occasionally participants are concerned (often unjustifiably so) about performance of their “weaker” hand, so would adopt a more conservative strategy by slowing down in practice trials.

## Experiment 1: Single-target pointing vs. two-target bisecting

The present study attempted to investigate right hemispheric contributions to visually-directed aiming by using a task which is strongly linked to right hemisphere specialization–bisection. Evidence from many clinical and experimental studies links poor performance on bisection of lines with right hemisphere damage. Paper and pencil line bisection frequently reveals neglect of left space in participants with RBD, as participants place their “mark” too far to the right (e.g., Schenkenberg et al., 1980; Milner et al., 1993). Additionally, it has been shown that in a task which was a visuomotor variant of line bisection, RBD patients who had recovered from hemispatial neglect performed more poorly than left-brain damaged patients. Thus, when the terminal endpoint for an aiming movements was defined by the perceived midpoint of two LEDs, RBD patients erred to the right, even though they were able to correct rightward deviations in the initial portion of reaches made directly to single LEDs (Goodale et al., 1990).

The aim of the present experiment was to determine whether it is possible to exaggerate the magnitude of the left hand RT advantage, therefore providing some evidence to support the hypothesis that this effect is driven by a right hemispheric specialization. In order to do so, we required participation in both a standard aiming task and a bisecting task, where the correct endpoints were co-incident in both. If accurate performance in bisection is more reliant on the right hemisphere than in single-target pointing, a left hand advantage in RT should be stronger in this condition. A second factor manipulated was the visibility of the hand during the reaching movement (also see Carson et al., 1992). Some studies have suggested that proprioception/kinesthesis may rely more heavily on right hemispheric systems (e.g., Guiard et al., 1983; Carson et al., 1990). If this hypothesis is correct, then any attenuation of right hand advantages in bisecting may be exaggerated in hand-invisible reaching.

## Methods

### Participants

Fourteen strongly right-handed males were tested. These volunteers were research assistants, graduate students and senior undergraduates from the University of Western Ontario. Participants completed a nine-item handedness questionnaire (a modified version of the Edinburgh Handedness Inventory; Oldfield, 1971) and were included in the study only if they performed all nine actions with their right hand. Participants ranged in age from 19 to 30 years (mean = 24.5).

### Procedure

Participants were required to reach quickly and accurately toward targets under two different hand visibility conditions, run on separate days; one in which the reaching limb was visible and the other in which the limb was not visible. Session was counterbalanced. Both hands were tested on each day, and the order of hand and task was also counterbalanced.

Participants pointed to single targets, or “bisected” two targets, in 30-trial blocks. Target light-emitting diodes (LEDs; red; 0.25°) were embedded in a Styrofoam wedge, centered 2 cm from the table surface, angled toward the participant's eyes, and covered in black speaker cloth (such that the location of LEDS was not visible until they were illuminated individually or in pairs). During a session, participants wore a black long-sleeved t-shirt and a black glove on the reaching limb (in order to eliminate as much as possible any visual cues from the limb during hand-invisible reaching). All calibration and test trials were performed while in a chinrest, angled to provide optimal viewing of the targets in the wedge. Small, infrared-emitting diodes (IREDs) were attached with Velcro to the tip and the base of the index finger on the glove. The three-dimensional locations of these diodes during calibration and test trials were recorded at 100 Hz using an opto-electronic recording system (WATSMART, Northern Digital, Inc.).

After collection of five calibration trials (where participants were allowed to adjust endpoint position to make perfect reaches to continuously-illuminated LEDS), participants were required to reach quickly and accurately to each presented LED target and to remain in their initial landing position until instructed to return to the start position and await the next trial. Participants were told that targets could appear anywhere on the target wedge in front of them, but were not told how many different targets would appear. Five different target positions were used (far left, near left, center, near right and far right, each 6 cm away from the adjacent target). The central target was located 32 cm in front of the start position, and the two most peripheral targets were 34 cm away from the start position (21.5° from the central target). Each target appeared six times, in a pre-determined, pseudo-random sequence.

**Figure 1:**
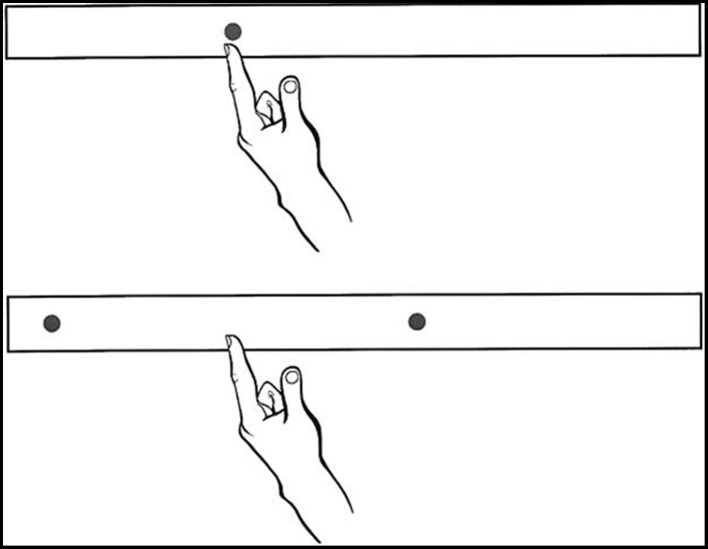
**Schematic representation of the pointing and bisecting conditions of experiment 1**. Five targets/midpoints were used, equally spaced by 6 cm from a midline target/midpoint. Adapted from Goodale et al. (1990).

For bisecting, participants were instructed to reach quickly and accurately to the midpoint between two simultaneously illuminated targets. The two LEDs for any particular bisecting trial were positioned 12 cm apart, and their true midpoints were located at the same positions as the five pointing targets (see Figure 1).

### Data analysis

After data collection, raw WATSMART files were converted to three-dimensional coordinates and filtered at 7-Hz with a second-order Butterworth filter. These filtered files were used to compute peak velocity (cm/s), movement onset time and movement duration (both in ms), and two different measures of endpoint accuracy (relative to the position of the fingertip LED specified by the calibration trial for that particular target/endpoint).

Each dependent measure was analyzed using three factor repeated measures analysis of variance, using the Geiser–Greenhouse adjustment of the degrees of freedom (for violations of homogeneity of covariance in repeated measures designs) when appropriate (Kirk, 1982; Tabachnick and Fidell, 1989). Significant interactions were explored using a simple main effects procedure (Kirk, 1982). The main effects and interactions relevant to hand differences in RT will be the main focus of the rest of this paper (see Supplementary Materials for statistical analyses of the other dependent measures in this and the other two experiments).

### Results

**Figure 2:**
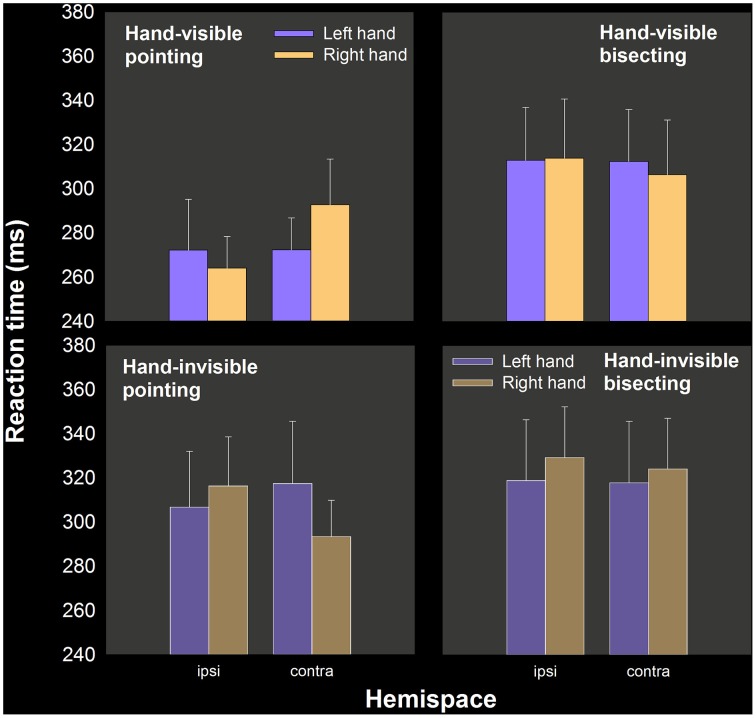
**Mean RT as a function of hand, hand visibility, hemispace, and task, experiment 1**. A lower left hand RT, relative to the right hand, was only obtained in hand-invisible bisecting, but this difference was not statistically significant.

The principal aim of the study was to see if left hand RT advantages in pointing would be increased by the theoretically more “right hemispheric” bisecting task relative to single target pointing. Table 1 includes mean RT as a function of hand, task and visual feedback condition. Figure 2 show these means separately for ipsilateral and contralateral hemispace (see Carey et al., 1996; Carey and Otto-de Haart, 2001). In only one of the four comparisons (two tasks × two hand visibility conditions) is the left hand even marginally quicker than the right. In fact, none of these differences in RT are statistically significant, even as assessed by 1-tailed paired samples *t*-tests: pointing: *t*_(13)_ = 0.215 and *t*_(13)_ = −0.634; bisecting: *t*_(13)_ = −0.475, and *t*_(13)_ = 0.702.

**Table 1:** **Mean RT (ms) as a function of task, hand and hand visibility**.

**Pointing**					**Bisecting**			
**Visible**		**Invisible**			**Visible**		**Invisible**	
**Mean**	**SEM**	**Mean**	**SEM**		**Mean**	**SEM**	**Mean**	**SEM**
269.3	13.4	300.2	17.6	**Right**	310.2	24.5	321.7	21.5
272.2	17.4	307.2	24.1	**Left**	312.7	23.5	312.9	25.9
−**2.9**		−**7**		**Diff (R–L)**	−**2.5**		+**8.8**	

*SEM = standard error of the mean. A positive difference score (bottom row) indicates a numerical left hand RT advantage*.

We also wanted to examine the proportions of these samples who show numerical left hand RT advantages. Even though these effects are small in neurologically-intact participants, if they are related to cerebral asymmetries in attentional/visuospatial processes then the majority of any right handed sample should show them (see Carey and Johnstone, 2014 for further discussion of the relevance of proportions for neuropsychological experiments comparing right- and left-handed participants). Of course, what precise proportion of dextral people who are right brain dominant for attentional of visuospatial function is not well-established, although see Cai et al. (2013) for some relevant data from fMRI. If one assumes complementary specialization of the right hemisphere when an individual is left hemisphere dominant for speech and language functions, then the proportion should be as high as 90–95%.

As suggested by the means, only one of the four conditions resulted in a majority proportion of the sample having left hand RT numerically smaller than right hand RT, which was in hand-invisible bisecting (0.64; hand-visible pointing = 0.50, hand-invisible pointing = 0.50, and hand-visible bisecting = 0.50).

### Discussion

These data are fairly easy to interpret in terms of left hand RT effects. In spite of the fact that our pointing task did not result in the often obtained left RT advantage, there is no evidence for bisecting shifting left hand RTs lower in relative terms. Numerically, at least, RTs did not favor the right hand in hand-invisible bisecting, but this shift relative to hand-invisible pointing was not statistically significant, even with rather liberal one-tailed t-testing. The proportions who show left hand RT advantages did change slightly in one bisecting condition (but the sample size in this experiment is rather small for this sort of proportional analysis).

There is little evidence to suggest what conditions tend to favor RT advantages for the left hand (after all that is what these experiments were designed to ascertain), therefore occasionally null findings in a control condition such as pointing in the present study, will limit the usefulness of any attempt to manipulate hand differences in RT. Unfortunately, this one interesting measure which usually favors the left hand of the right hander, is not obtained in every experiment.

Undeterred by this first attempt, with a new sample of right handers we attempted a somewhat different type of manipulation, targeted more directly at the *localization* demands of a manual aiming task.

## Experiment 2: Manipulating localization demands by increasing the number of target locations

One sensible way to try and quantify the relative contribution of the right hemisphere to motor control would be to keep the task focussed on the spatial localization of targets for the production of rapid movements. Localization refers to a diverse set of processes which allow for specifying the location of an external target to some sort of egocentric or body-centered representation (Bock, 1986; Miller, 1996). The evidence for a right hemisphere advantage for the localization of targets (Kimura, 1969), perhaps in relatively early stages of movement planning (Carson et al., 1992) motivated this second experiment. Here, we varied the visuospatial demands of the task by manipulating target uncertainty, while requiring identical motor responses (as in experiment 1). If a left-hand advantage for movement onset reflects right-hemispheric specialization for target localization, the expectation was that increasing the spatial uncertainty of the task by increasing the number of target locations should result in an interaction between hand used and target number of targets. We expected the largest left hand RT advantage occurring in the block with the greatest number of possible target positions. In addition, RT advantages should be decreased in the 2 target condition, relative to the intermediate 6 target condition, if target localization demand predicts left hand RT advantages.

## Methods

### Participants

Participants were 22 volunteers, 12 females and 10 males, mainly undergraduate and postgraduate students from the University of Aberdeen, ranging in age from 18 to 41 years (mean = 26.6, SD = 6.9). They were self-declared strong right-handers, verified by a 9-item handedness inventory (a modified version of the Edinburgh Handedness Inventory; Oldfield, 1971). All participants were naïve as to the purpose of the experiment and took part in two test sessions, one in which the right hand was tested and the other in which the left hand was tested, run on separate days.

### Procedure

**Figure 3:**
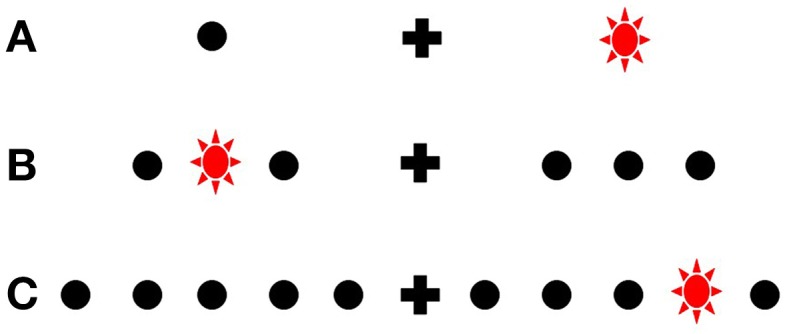
**Schematic representation of target uncertainty in experiment 2**. Only two possible target positions could be illuminated in Block **(A)**, relative to the 10 possible in Block **(C)**. Note how the each array is centered equivalently relative to fixation [i.e., middle targets in **(B)** and **(C)** are aligned with the only target in each hemispace in **(A)**]. Drawing not to scale.

A 60 Hz MacReflex three camera motion capture system was used, coupled with a bespoke light emitting target board controlled by an adjacent PC. The participants were given six blocks of trials in which the number of targets used were varied. Each block's targets were symmetrical, and centered 15 cm (14.6°) to the left and right of a midline fixation point (the two targets used in block A were at these two positions; see Figure 3). In block B, six targets were used; inner targets were 9.8° from fixation and the outer were at 19.5°. In block C, 10 targets were used, equidistant, at 4.9° at most medial to the most lateralized at 24.2°.

The targets presented in each block appeared randomly. The blocks were run in an ABCCBA or CBAABC order, to counterbalance for any potential practice or fatigue effects. The two middle blocks (which both had either 10 targets or 2 targets) were separated by a brief delay. Half of the participants began with block A, while the other half began with block C.

In the test session a total of 144 trials were run for each hand. In each block 4 pointing movements were required, in random order, to each stimulus target used. Practice trials (one movement to each possible target) were provided in each of the first new blocks to familiarize the participant with the number and location, of the 2, 6, or 10 targets. All participants were also told the number of targets in each block verbally before the practice trials.

### Results

**Figure 4:**
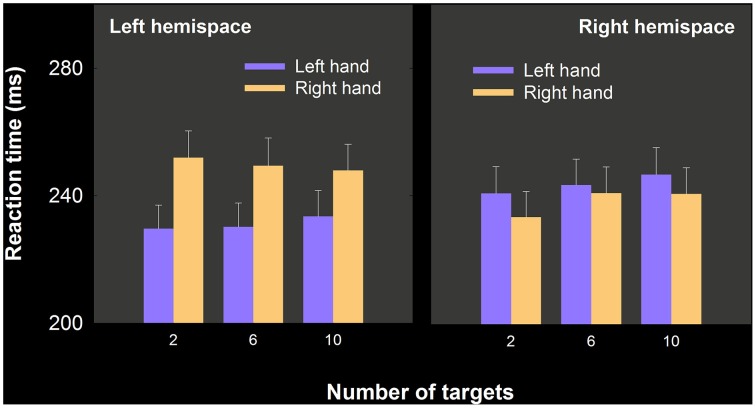
**Mean RT as a function of hand, number of targets and hemispace, experiment 2**. Left hand RT advantages were significant, depended on larger ipsilateral advantages than those obtained in the right hand, but did not interact with the number of targets. Note that in each panel, we have plotted ipsilateral (i.e., left hemispace, left hand; right hemsipace, right hand) movement means before contralateral movement means.

We predicted an interaction between number of targets and hand, such that RT differences that favor the left hand should have been largest in the 10-target condition and smallest in the 2-target condition. The data, shown separated for left and right hemispace, are depicted in Figure 4.

A three way repeated measures analysis of variance with hand, number of targets, and hemispace was performed. Overall the hands did not differ in RT [*F*_(1, 20)_ = 3.75, *p* = 0.067], ipsilateral movements were initiated more quickly than contralateral movements [*F*_(1, 20)_ = 27.57, *p* < 0.001]. No other effects were statistically significant, including the Hand × Number of targets × Hemispace [*F*_(2, 40)_ = 3.132, *p* = 0.054]. Nevertheless given our directional prediction we ran three paired samples *t*-tests on the 2, 6, and 12 target blocks comparing right hand–left hand RT. The left hand RT advantage was only statistically significant for the 6 target conditions [*t*_(21)_ = 2.35, *p* < 0.02]. We also compared the proportion of the sample who showed a *numerical* left hand advantage in the 2, 6, and 10 target conditions. The resulting proportions were 0.67, 0.76, and 0.71.

A summary of results for other dependent measures appears in the Supplementary Materials.

### Discussion

As in experiment 1, there was little evidence for any exaggeration of left hand RT advantages as target numbers increased from 2 to 6 to 10. In this experiment, unlike the previous one, there was at least a possibility for exaggeration or attenuation of the left hand RT effect, as most of our participants (76%) had numerical left hand RT advantages in the “intermediate” 6-target block (which in some sense is the control condition in this experiment). Nevertheless, the mean RT effect did not increase or decrease significantly across blocks, and the proportion of the sample who have numerical left hand advantages was virtually unchanged in 2-, 6-, and 10-target conditions.

Of course, targets were restricted to placement within a horizontal array, which may not have taxed systems that normally localize with eye, head and hand in a multidimensional world. Furthermore, although more target uncertainty was introduced, theoretically the attentional demands of the 2, 6, and 10 target conditions may not have differed by much; the horizontal extent of the space which may have contained targets for any block varied from 30° (2 target blocks) to 46° (6 target blocks). These two horizontal extents are well within the binocular visual fields, and may not have differed sufficiently in terms of the extent of space to be monitored for potential targets in a speeded aiming task. In fact, while we were designing this task we became well-aware of how different distances and or different spatial resolutions are necessary to vary target number—it was difficult to know how to trade these factors off with one another in the absence of any strong data on left hand RT mechanisms. In any case, this kind of thinking about attention in reaching led us to our final experiment, where we used a manipulation coupled to two somewhat distinct *attentional* mechanisms, both linked with right-hemisphere specialization, which may account for the left hand RT effect.

An additional analysis of a subset participants who showed left hand RT advantages overall, also provided no support for the hypothesis that target number influenced the magnitude of the left hand RT advantage.

## Experiment 3: Fixation-target “gap” vs. “no-gap” pointing

In the final experiment, we attempted a manipulation directed toward a more attentional explanation of the left hand RT effect. Right hemisphere specialization for attentional systems has been suggested for some time, from studies of patients with hemispatial neglect (Brain, 1941; De Renzi, 1982; Danckert and Ferber, 2006) and neurotypical participants (e.g., Gitelman et al., 1999; Jewell and McCourt, 2000; Rushworth et al., 2001; Mattingley et al., 2004; Shulman et al., 2010; Voyer et al., 2012)^1^. Of course, in single-target aiming, two different types of attention may play roles in facilitating rapid responses. First, generalized alertness or vigilance (Marrocco et al., 1994), could be facilitated by preparing to use the left hand, largely controlled and monitored by motor, premotor and somatosensory networks of the right hemisphere (for evidence linking generalized alertness to the right hemisphere, see Posner and Peterson, 1990; Robertson et al., 1998). An alternative attentional mechanism might be related to a more spatially—selective process such as visual orienting to a target (Posner and Peterson, 1990; Petersen and Posner, 2012).

For this study, we chose a manipulation which has requirements related to both types of attentional component—the “gap effect” (Saslow, 1967). This effect refers to facilitated RTs for targets when a short delay (typically 100–200 ms) between fixation offset and target onset is introduced. Although described initially in a two-target saccadic eye movement paradigm (Saslow, 1967; Fischer and Ramsperger, 1984) a manual gap effect has also been identified, although there is some debate over whether or not the effects are carried over from saccadic facilitation (Bekkering et al., 1996). Perhaps coincidentally, the magnitude of the manual gap effect is typically around 15–20 ms (Reuter-Lorenz et al., 1991; Bekkering et al., 1996; Fendrich et al., 1999), which is not disproportionately larger than the left hand RT advantages in reaching experiments. What was crucial for our purposes was that there is some evidence that the gap effect results from *both* the general alerting effect of fixation offset (Dorris and Munoz, 1995; which takes some time to manifest itself) and from a spatial orienting/facilitation effect (e.g., attention is released from fixation which can now be allocated in the direction of a manual/saccadic target; Kingstone and Klein, 1993; Pratt et al., 2000; Rolfs and Vitu, 2007). In other words, this study was a first pass at an attention explanation of the left hand RT advantage, which we intended to explore if successful using manipulations from the gap effect literature designed to fractionate the alerting and orienting components.

Interestingly, there are some indications of a hemispatial asymmetry in the manual gap effect. Lünenburger et al. (2000) found a slightly larger manual gap effect when right-handers reached toward the right side of space, compared to equivalent left-sided reaches. However, as the left hand was not examined in that experiment, the conclusions that can be drawn from this finding are limited. Gomez and colleagues also found larger gap effects when dextrals had to react to targets appearing in their right visual field (Gómez et al., 1998). However, this study tested only choice reaction times (pressing left mouse button for a left target and the right mouse button for a right target), rather than manual localization, as was required here.

The current experiment includes data from three separate gap effect studies performed by GB, HCD, and DPC, which differed slightly in precise methods but all required: (1) right-handed participants to reach in gap and no gap (fixation offset coincident with target onset) conditions; (2) target arrays that were balanced with respect to the participant's midline (i.e., half in each hemispace), and (3) separate blocks of left and right hand unimanual reaches, made as quickly (and accurately) as possible.

## Methods

### Participants

A total of 67 participants were tested over the course of the 3 experiments (26 in study 1, 21 in study 2 and 20 in study 3). The mean age of the samples was 22.0 years, SD = 2.83. All participants had normal or corrected to normal vision. All participants were dextral, with strength of hand preference measured by a modified version of the Waterloo Handedness Questionnaire (WHQ; Steenhuis and Bryden, 1989; mean = 26.85/30; SD = 3.48). Participants were naïve to the hypothesis (including the inclusion of the temporal gap) and gave informed consent prior to testing, with all procedures approved by the Ethics Committee of the School of Psychology at the University of Aberdeen.

### Procedure

Each participant was tested individually in a single session in a darkened room to minimize infrared reflections and allow for easy detection of peripheral targets. The participant sat (head free) on a height-adjustable chair in front of a bespoke horizontal light emitting diode (LED) grid board. Their index finger was then placed upon the starting location, marked by a tactile Velcro pad on the near side of the board in line with the fixation point. Prior to commencement of each trial, the experimenter gave an auditory “pre-start” cue (“Ready…”) and started the trial with an audible key press. The central fixation light appeared (which the participant was required to fixate) for a short duration and was then extinguished, followed by either the immediate appearance of one of the targets (“no gap” condition) or a temporal gap (200 ms for the Studies 1 and 2, 160 ms for Study 3) before the appearance of a target (“gap” condition).

An infrared reflective marker was attached to the index finger of the participant's reaching hand, the position of which was monitored with either a two-camera MacReflex motion analysis system, recording at 60 Hz (Studies 1 and 3) or an Optotrak motion analysis system, recording at 200 Hz (Study 2). The camera positions were calibrated prior to each testing session. Studies 1 and 2 required 200 trials, while Study 3 required 192 trials. Four (Study 1), six (Study 2), and eight (Study 3) different targets were presented.

### Results

**Figure 5:**
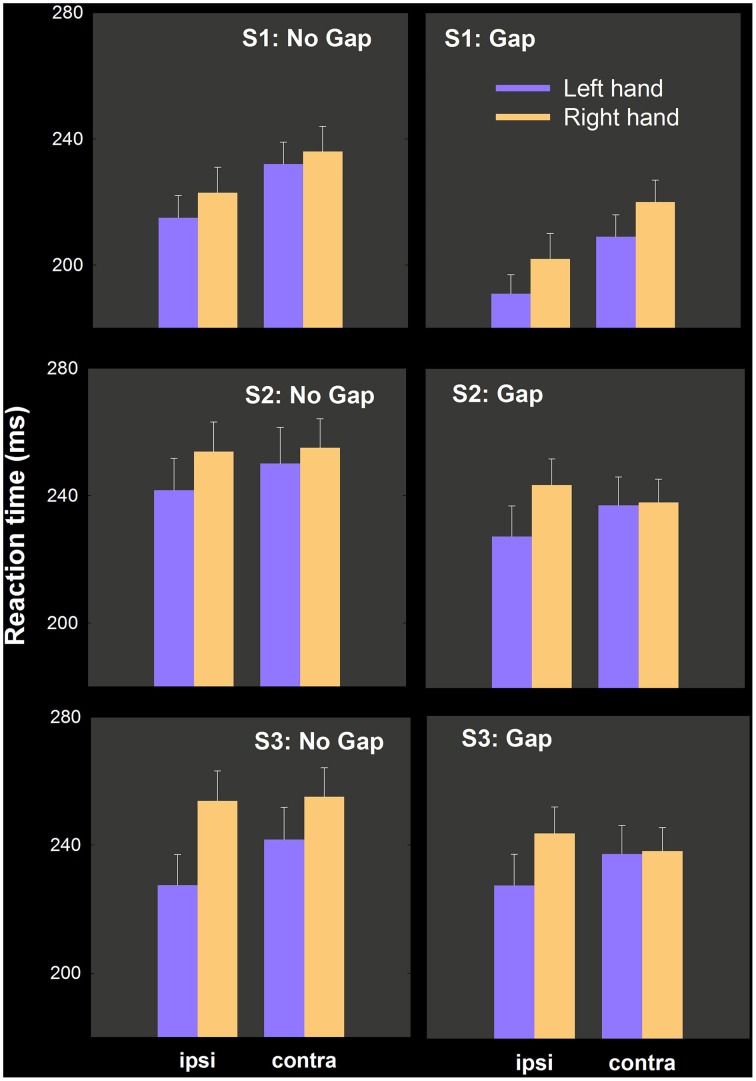
**Mean RT as a function of condition (no gap, gap), hand and hemispace**. Our prediction was that left hand RT advantages would be enhanced in gap conditions relative to no gap conditions. Significant hand differences in no gap conditions were not modulated statistically by the introduction of a fixation-target gap.

We report Task (no gap, gap) × hand (right, left) × hemispace (ipsilateral, contralateral) ANOVAs for RT in each study first, and combine all in an omnibus analysis with all 67 participants. Mean RTs as a function of this factor are depicted separately for each study in Figure 5.

#### Study 1

Main effects of Task [*F*_(1, 25)_ = 34.16, *p* < 0.001], Hand [*F*_(1, 25)_ = 15.24, *p* < 0.002] Hemispace [*F*_(1, 25)_ = 56.72, *p* < 0.001] are explained by quicker movement initiation by the left hand (8 ms), in gap trials (22 ms), and in ipsilateral hemispace (16 ms). No higher order interactions involving hand and task were obtained, in spite of a numerically larger hand difference in gap (11 ms) vs. no gap (6 ms) conditions in the predicted direction. These means and associated variance estimates are illustrated in the top row of Figure 5. As in experiment 1 above, we calculated the proportion of the sample who have numerically smaller left hand RT: in no gap, 18/26 (0.69); in gap, 22/26 (0.85).

#### Study 2

The same three factor repeated measures ANOVA uncovered significant effects of Task [*F*_(1, 20)_ = 8.69, *p* < 0.009; gap 11 ms quicker than no gap], Hand [*F*_(1, 20)_ = 8.09, *p* < 0.02; left hand quicker by 14 ms] as well as Task by Hand [*F*_(1, 20)_ = 9.73, *p* < 0.006] and Hand by Hemispace [*F*_(1, 20)_ = 7.01, *p* < 0.02] interactions. The three way interaction between Task, Hand, and Hemispace was not significant [*F*_(1, 20)_ = 0.14, N.S.] The relevant means and variance estimates appear in the middle row of Figure 5.

The Task by Hand interaction may be due to a significant drop in RT in the right hand [15 ms; *t*_(20)_ = 3.26, *p* < 0.005] but not in the left hand [2 ms; *t*_(20)_ = 1.16, N.S.] in gap relative to no gap conditions (contrary to expectations). The Hand by Hemispace interaction may be due to no significant hemispace effect for the right hand [−1.1. ms; *t*_(20)_ = −0.69, NS] while the left hand was significantly quicker in ipsilateral space [12 ms; *t*_(20)_ = 2.91, *p* < 0.01].

As in experiment 1 and study 1, we calculated the proportion of the sample who have numerically smaller left hand RT in each condition: in no gap, 17/21 (0.81); in gap, 16/21 (0.76).

#### Study 3

Main effects of Task [*F*_(1, 19)_ = 94.12, *p* < 0.001], Hand [*F*_(1, 19)_ = 6.49, *p* < 0.03], Hemispace [*F*_(1, 25)_ = 4.95, *p* < 0.04] are explained by quicker initiation by the left hand (10 ms), in gap trials (26 ms), and in ipsilateral hemispace (6 ms). As in study 1, no higher order interactions involving hand and task were obtained, in spite of a numerically larger hand difference in gap (14 ms) vs. no gap (8 ms) conditions in the predicted direction. These means and associated variance estimates are illustrated in the bottom row of Figure 5. As above, we calculated the proportion of the sample who have numerically smaller left hand RT: in no gap, 13/20; in gap, 13/20 (both = 0.65).

In summary, the RT differences between the hands are small, and for two of three studies 60 Hz recordings have relatively poor temporal resolution, at least on single trials (see General Discussion). We thought that given the completely repeated measures nature of all three studies, we could combine these datasets.

#### Omnibus analysis

The typical main effects of Task (19 ms; $\eta_{p}^{2}=0.56$), Hand (11 ms; $\eta_{p}^{2}=0.28$), and Hemispace (9 ms; $\eta_{p}^{2}=0.38$) are significant, as in the individual experiments, but of most relevance here are the two way interaction between Task and Hand [*F*_(1, 66)_ = 0.007, NS] and the three way interaction between Task, Hand, and Hemispace [*F*_(1, 66)_ = 0.748, NS]. These data suggest that adding a gap between fixation offset and target onset do not have any effects on the left hand RT advantage.

## General discussion

We report on three sets of studies where we attempted to increase or decrease left hand advantages in RT for visually-guided aiming movements. We used three different tasks to do so: two-target bisecting (linked to right hemispheric specialization), target uncertainty (as a proxy for visuo-spatial processing/localization) and the gap effect (linked to both vigilance and visuospatial orienting, both related to somewhat distinct but nevertheless right hemispheric circuitry). Our results provide very little evidence for any effect of these three manipulations on hand differences in RT.

In experiment 1, we managed to obtain one of those relatively rare aiming samples where left hand RT advantages were not found, which limited the scope for clear attenuation of such effects in bisecting. In experiment 2, quite reliable left hand RT advantages were found in our second sample of right handers, but these were little changed by increasing target number from 6 to 10 or decreasing target number from 6 to 2. In our final experiment, across three separate studies with different participants, there was little suggestion of increased hand differences in RT when comparing gap to no gap conditions. In addition to main effects and the hypothesized two way interaction between hand and task, we looked for evidence if hemispace moderated any differences. It didn't.

Our tasks may not have been optimized for “pushing” a right hemisphere lateralized mechanism for either attentional processes or for visuospatial analysis. In the former case, in all tasks participants waited (vigilantly we hope) for a single target which appeared in a relatively restricted horizontal meridian. Although in bisection two targets needed to be processed, a limited number of such pairs (5), of identical inter-target distances, may have allowed some participants to identify the limited number of response points, or to plan their movements relative to one of the two members of the pair, etc. With current stimulus generation/display capabilities there is no reason why bisection performance could not be required with varying pair sizes and orientations in space, which might tax any visuospatial mechanisms to a greater extent than our relatively simple stimulus display (which was designed for use with elderly participants; Goodale et al., 1990).

We have rather little data, surprisingly, which let us predict what aiming experiments left hand RT advantages are obtained in, vs. those in which they are not. There are suggestions that adding choice to RT experiments may typically elicit right hand advantages, but to our knowledge this type of manipulation hasn't been varied systematically, at least in the hand difference literature. Similarly, the importance of visually-guided reaching for left hand RT advantage has yet to be decisively established, relative to, simple RT in detection tasks, for example. One group claim that the left hand RT advantage in the same participants depends on actually making a reach to a target (Mieschke et al., 2001), while another group claims that it does not (Barthélémy and Boulinguez, 2002). These studies may be limited somewhat by their small number of targets and/or participants. They also approach their data sets quite differently in statistical terms. One of us is currently attempting a replication of these experiments with a larger sample. Our gap effect manipulation was designed as a first pass within this domain, but was unsuccessful.

Given the failure to find a left hand RT advantage in the pointing task of experiment 1, it could be useful to perform power calculations for informing sample size requirement in future tasks. In fact, such an estimate is difficult to calculate, given that hand is a repeated measure in these designs and the variance of the difference scores, as well as the correlation between right and left hand RT, are needed for the calculations (Dunlap et al., 1996; Morris and DeShon, 2002; Maxwell et al., 2008). These measures are not provided in published papers. In addition, we now prefer a point estimate/accuracy approach to sample size planning, as advocated by Kline (2005), Maxwell et al. (2008), and Cumming (2012). These techniques avoid questions of how large an effect size exists in the population. Instead, experimenters consider how large confidence intervals could be before a particular sample would become uninformative.

For these estimates of sample size for precision, we created an estimate of standard deviation of hand RT differences from the current five and an additional eight in-house studies, where variance of the difference scores was known in each. Using the techniques of Cumming (2012) we estimate that a sample size of at least 17 people is required, *on average*, to ensure that a 95% confidence interval surrounding a left hand RT advantage does not overlap with zero. To ensure that 99% of the time the confidence interval would never overlap with zero (what Cummings refers to as the “with assurance” calculation), a sample size of 28 is required (see Supplementary Materials for additional information and a figure depicting estimated CI size and sample size).

We have to acknowledge that the left hand RT advantage may not depend on its' privileged connections to the right hemisphere. In fact, it is equivalently parsimonious to consider the typical difference as *an increase* in right hand RT; it may be related to superior motor control capacity of the left hemisphere in most conventionally dominant dextrals, or may even be related to many years' experience of skilled sensorimotor activity related to drawing and writing with the preferred hand. Of course such experience might manifest itself in specialized networks of the left hemisphere, but these may depend on practice and experience. In any case, the suggestion that the hand difference is not related to innate processing pre-dispositions of the left or right cerebral hemisphere, is a testable one: quantify the same dependent measures in left-handed participants. Although most left handed people, like their right handed counterparts, are left hemisphere dominant for speech and language, the proportion is smaller in this group (roughly 70 vs. 95% in right handers; Rasmussen and Milner, 1977). If directional behavioral results (e.g., hand differences, ear advantages in dichotic listening, or visual field biases) depend on hemispheric asymmetry, they will mimic the direction of difference in dextrals (left hand RT < right hand RT; right ear syllable score greater than left ear syllable score, etc.). The magnitude of the effect, however, will be reduced. This reduction would follow a small proportion of the adextral group having bilateral or reversed cerebral dominance (Carey and Johnstone, 2014)^2^.

We hesitate at this stage to avoid the ubiquitous but often trite suggestion that “further research is needed.” The more interesting question is what kind of research is needed (or if any indeed is required—this effect, when obtained is quite small; approximately 7 ms on average, based on 13 separate hand difference studies in our laboratory.

First, we would suggest that any sort of speed-accuracy trade off in the left hand relative to the right be systematically eliminated as a major factor in left hand RT advantages (such a suggestion has been made in the reciprocal tapping literature, for example; see Carson, 1992, for review). We already know that if there is such a trade-off, it would have to do with pre-movement processing, as the dominant hand of the right hander is faster and more accurate, once it is off the mark. Our accuracy data of experiment 1 (see Supplementary Materials) suggest that this is unlikely, but perhaps this hypothesis needs to be eliminated more systematically, within subject, on a trial by trial basis. In fact, in hand-visible reaching, accuracy differences tend to be quite small, and are often restricted to increased variability in the left hand of the right hander (e.g., Roy and Elliott, 1989; Carson et al., 1990). Often we don't bother to measure it, and restrict our analysis instead to speed-related dependent measures. In any case, we certainly see little evidence for speed accuracy trade-offs across participants, in experiment 1 or in other experiments we have performed.

Another approach to addressing mechanisms accounting for left hand RT advantages might consider the distributions of left and right handed movement RTs in participants who show these effects robustly, and then characterize them in much more detail than the usual mean/ANOVA approach that many scientists in this domain have favored. For example, are the distributions shifted by approximately 8–10 ms, or is there a small population of very fast left-handed movements, roughly analogous to “express saccades” seem in the saccadic gap literature (e.g., Wenban-Smith and Findlay, 1991)?

Carson (1996) has suggested that the spatial demands of reaching may be relatively impervious to these types of manipulations of the stimulus, suggested by several experiments by him and his colleagues which fail to affect the left hand RT advantage (as well as the three experiments reported here). His later comments on left RT advantages introduced the idea of “spatial parametrization,” integrating information about parts of the body in a feedforward manner for comparison to elements of the environment (Carson et al., 1995; Carson, 1996). The idea seems similar to the literature on the coordinate transformations required to get from a retinal representation of target position to a hand- or arm-centered scheme (reviewed in Carey, 2004; Crawford et al., 2011). More details on this sort of idea are probably necessary to generate testable hypotheses for reaching experiments. The literature on coordinate transformations has grown considerably since the 1990s, but it is not obvious to us how some of these computations would map neatly onto ideas about the right hemisphere. In fact, different scientists have argued, based on quite distinct sensorimotor tasks, that feedforward (e.g., Meyer et al., 1988; Adam et al., 2010) or feedback processes (e.g., Roy et al., 1994) favor left hemisphere-right hand sensorimotor control.

The “face validity” of linking left hand RT advantages to some sort of right hemispheric process still has some appeal. If these hand differences (RT, accuracy, peak velocity, duration) are related to relatively innate cerebral specializations, then predictions can be made about the same dependent measures when assessed in visually-guided reaching movements of left handers. In other words, if these effects are strongly related to cerebral asymmetries, then many left handers (roughly 70% are left hemisphere dominant for speech and language) should behave like right handers, literally (left hand RT advantages, right hand duration and peak velocity advantages, etc.). There is some evidence for this state of affairs (Boulinguez et al., 2001a,b) or at least for weakened (but not reversed) asymmetries in a group of left handers (Goodale, 1990). The discrepancies may be partially resolved by a more detailed description of both the depth and breadth of hand preferences in both right and left handers, as well as consideration of the proportions of individuals in each group which show any directional effect (Carey and Johnstone, 2014).

### Conflict of interest statement

The authors declare that the research was conducted in the absence of any commercial or financial relationships that could be construed as a potential conflict of interest.

## Footnotes

^1^Surprisingly few studies report left visual field advantages in neurotypical participants for visual search and Posner-like cueing tasks; (see Palmer and Tzeng, 1990; Evert et al., 2003; Michael and Ojéda, 2005; Poynter and Roberts, 2012, for examples, caveats and analysis).

^2^Remarkably little is known, in adextrals, about asymmetries that tend to favor the right hemisphere in dextrals, beyond face processing (although see Elias and Bryden, 1998. A second paper on prosody has a promising title suggesting a comparison of left and right handers. Sadly it has a sample size of two in each group: Perry et al., 2001).

## References

[B1] Adam J. J. Müskens R. Hoonhorst S. Pratt J. Fischer M. H. (2010). Left hand, but not right hand, reaching is sensitive to visual context. Exp. Brain Res. 203, 227–232. 10.1007/s00221-010-2214-6 20300930 PMC2862955

[B2] Bagesteiro L. B. Sainburg R. L. (2002). Handedness: dominant arm advantages in control of limb dynamics. J. Neurophysiol. 88, 2408–2421. 10.1152/jn.00901.2001 12424282 PMC10709816

[B3] Barthélémy S. Boulinguez P. (2002). Orienting visuospatial attention generates manual reaction time asymmetries in target detection and pointing. Behav. Brain Res. 133, 109–116. 10.1016/S0166-4328(01)00446-6 12048178

[B4] Bekkering H. Pratt J. Abrams R. A. (1996). The gap effect for eye and hand movements. Percept. Psychophys. 58, 628–635. 8934692 10.3758/bf03213095

[B5] Bock O. (1986). Contribution of retinal versus extraretinal signals towards visual localization in goal-directed movements. Exp. Brain Res. 64, 476–482. 3803485 10.1007/BF00340484

[B6] Boulinguez P. Nougier V. Velay J. L. (2001a). Manual asymmetries in reaching movement control. I: study of right-handers. Cortex 37, 101–122. 10.1016/S0010-9452(08)70561-6 11292156

[B7] Boulinguez P. Velay J. L. Nougier V. (2001b). Manual asymmetries in reaching movement control. II: study of left-handers. Cortex 37, 123–138. 10.1016/S0010-9452(08)70562-8 11292158

[B8] Brain W. R. (1941). Visual disorientation with special reference to lesions of the right cerebral hemisphere. Brain 64, 244–272. 19992404 10.1177/003591574103401212 PMC1998360

[B9] Cai Q. Van der Haegen L. Brysbaert M. (2013). Complementary hemispheric specialization for language production and visuospatial attention. Proc. Natl. Acad. Sci. U.S.A. 110, E322–E330. 10.1073/pnas.1212956110 23297206 PMC3557046

[B10] Carey D. P. Hargreaves E. L. Goodale M. A. (1996). Reaching to ipsilateral or contralateral targets: within-hemisphere visuomotor processing cannot explain hemispatial differences in motor control. Exp. Brain Res. 112, 496–504. 9007551 10.1007/BF00227955

[B11] Carey D. P. Johnstone L. T. (2014). Quantifying cerebral asymmetries for language in dextrals and adextrals with random-effects meta analysis. Front. Psychol. 5:1128. 10.3389/fpsyg.2014.01128 25408673 PMC4219560

[B12] Carey D. P. Otto-de Haart E. G. (2001). Hemispatial differences in visually guided aiming are neither hemispatial nor visual. Neuropsychologia 39, 885–861. 10.1016/S0028-3932(01)00036-7 11516441

[B13] Carey D. P. (2004). Neuropsychological perspectives on sensorimotor integration, in Functional Brain Imaging of Visual Cognition. Attention and Performance XX, eds Nancy K. John D. Carlo U. (Cambridge, MA: The MIT Press), 481–502.

[B14] Carson R. G. Chua R. Goodman D. Byblow W. D. Elliott D. (1995). The preparation of aiming movements. Brain Cogn. 28, 133–154. 7546669 10.1006/brcg.1995.1161

[B15] Carson R. G. Elliott D. Goodman D. Dickinson J. (1990). Manual asymmetries in the reproduction of a 3-dimensional spatial location. Neuropsychologia 28, 99–103. 2314568 10.1016/0028-3932(90)90090-b

[B16] Carson R. G. Elliott D. Goodman D. Thyer L. Chua R. Roy E. A. (1993b). The role of impulse variability in manual-aiming asymmetries. Psychol. Res. 55, 291–298.

[B17] Carson R. G. Goodman D. Chua R. Elliott D. (1993a). Asymmetries in the regulation of visually guided aiming. J. Mot. Behav. 25, 21–32. 12730038 10.1080/00222895.1993.9941636

[B18] Carson R. G. Goodman D. Elliott D. (1992). Asymmetries in the discrete and pseudocontinuous regulation of visually guided reaching. Brain Cogn. 18, 169–191. 1575975 10.1016/0278-2626(92)90077-y

[B19] Carson R. G. (1992). Visual feedback processing and manual asymmetries: an evolving perspective. Advances in Psychology, 85, 49–65.

[B20] Carson R. G. (1996). Putative right hemisphere contributions to the preparation of reaching and aiming movements. Hand preference and performance in skilled and unskilled activities, in Manual Asymmetries in Motor Performance, eds Elliott D. Roy E. A. (Boca Raton, FL: CRC Press), 159–172.

[B21] Chua R. Carson R. G. Goodman D. (1992). Asymmetries in the spatial localization of transformed targets. Brain Cogn. 20, 227–235. 1449755 10.1016/0278-2626(92)90017-g

[B22] Crawford J. D. Henriques D. Y. Medendorp W. P. (2011). Three-dimensional transformations for goal-directed action. Ann. Rev. Neurosci. 34, 309–331. 10.1146/annurev-neuro-061010-113749 21456958

[B23] Cumming G. (2012). The New Statistics: Effect Sizes, Confidence Intervals, and Meta-Analysis. New York, NY: Taylor and Francis.

[B24] Danckert J. Ferber S. (2006). Revisiting unilateral neglect. Neuropsychologia 44, 987–1006. 10.1016/j.neuropsychologia.2005.09.004 16300805

[B25] De Renzi E. (1982). Disorders of Space Exploration and Cognition. New York, NY: John Wiley and Sons.

[B26] Dorris M. C. Munoz D. P. (1995). A neural correlate for the gap effect on saccadic reaction times in monkey. J. Neurophysiol. 73, 2558–2562. 7666161 10.1152/jn.1995.73.6.2558

[B27] Dunlap W. P. Cortina J. M. Vaslow J. B. Burke M. J. (1996). Meta-analysis of experiments with matched groups or repeated measures designs. Psychol. Methods 1, 170–177.

[B28] Elias L. J. Bryden M. P. (1998). Footedness is a better predictor of language lateralisation than handedness. Laterality 3, 41–52. 15513074 10.1080/713754287

[B29] Elliott D. Chua R. Pollock B. J. (1994). The influence of intermittent vision on manual aiming. Acta Psychol. 85, 1–13. 8165920 10.1016/0001-6918(94)90016-7

[B30] Elliott D. Roy E. A. (1996). Manual Asymmetries in Motor Performance. Boca Raton, FL: CRC Press.

[B31] Elliott D. Roy E. A. Goodman D. Carson R. G. Chua R. Maraj B. K. V. (1993). Asymmetries in the preparation and control of manual aiming movements. Can. J. Exp. Psychol. 47, 570–589.

[B32] Evert D. L. McGlinchey-Berroth R. Verfaellie M. Milberg W. P. (2003). Hemispheric asymmetries for selective attention apparent only with increased task demands in healthy participants. Brain Cogn. 53, 34–41. 10.1016/S0278-2626(03)00207-0 14572500

[B33] Fendrich R. Demirel S. Danziger S. (1999). The oculomotor gap effect without a foveal fixation point. Vision Res. 39, 833–841. 10341969 10.1016/s0042-6989(98)00164-3

[B34] Fischer B. Ramsperger E. (1984). Human express saccades: extremely short reaction times of goal directed eye movements. Exp. Brain Res. 57, 191–195. 6519226 10.1007/BF00231145

[B35] Fisk J. D. Goodale M. A. (1985). The organization of eye and limb movements during unrestricted reaching to targets in contralateral and ipsilateral visual space. Exp. Brain Res. 60, 159–178. 4043274 10.1007/BF00237028

[B36] Fisk J. D. Goodale M. A. (1988). The effects of unilateral brain damage on visually guided reaching: hemispheric differences in the nature of the deficit. Exp. Brain Res. 72, 425–435. 3224652 10.1007/BF00250264

[B37] Gitelman D. R. Nobre A. C. Parrish T. B. LaBar K. S. Kim Y. H. Meyer J. R.. (1999). A large-scale distributed network for covert spatial attention. Brain 122, 1093–1106. 10356062 10.1093/brain/122.6.1093

[B38] Goldenberg G. (2013). Apraxia: The Cognitive Side of Motor Control. Oxford: Oxford University Press. 10.1016/j.cortex.2013.07.016 24144066

[B39] Gómez C. Millán S. Atienza M. Aguilar-Bravo H. Vázquez M. Delinte A. (1998). The gap effect during visual and auditory stimulation using manual responses. Biol. Psychol. 47, 77–96. 9505135 10.1016/s0301-0511(97)00022-7

[B40] Goodale M. A. Milner A. D. Jakobson L. S. Carey D. P. (1990). Kinematic analysis of limb movements in neuropsychological research: subtle deficits and recovery of function. Can. J. Psychol. 44, 180–195. 2200594 10.1037/h0084245

[B41] Goodale M. A. (1988). Hemispheric differences in motor control. Behav. Brain Res. 30, 203–214. 3048307 10.1016/0166-4328(88)90149-0

[B42] Goodale M. A. (1990). Brain asymmetries in the control of reaching, in Vision and Action: The Control of Grasping, ed Goodale M. (Norwood, NJ: Ablex Publishing), 14–32.

[B43] Guiard Y. Diaz D. Beaubaton D. (1983). Left-hand advantage for right-handers for spatial constant error: preliminary evidence in a unimanual ballistic aimed movement. Neuropsychologia 21, 111–115. 10.1016/0028-3932(83)90106-9 6843811

[B44] Haaland K. Y. Harrington D. (1989). The role of the hemispheres in closed loop movements. Brain Cogn. 9, 158–180. 2923708 10.1016/0278-2626(89)90027-4

[B45] Haaland K. Y. Harrington D. (1996). Hemispheric asymmetry of movement. Curr. Opin. Neurobiol. 6, 796–800. 9000021 10.1016/s0959-4388(96)80030-4

[B46] Jewell G. McCourt M. E. (2000). Pseudoneglect: a review and meta-analysis of performance factors in line bisection tasks. Neuropsychologia 38, 93–110. 10.1016/S0028-3932(99)00045-7 10617294

[B47] Kimura D. Archibald Y. (1974). Motor functions of the left hemisphere. Brain 97, 337–350. 4434181 10.1093/brain/97.1.337

[B48] Kimura D. (1969). Spatial localization in left and right visual fields. Can. J. Psychol. 23, 445–458. 5366960 10.1037/h0082830

[B49] Kimura D. (1993). Neuromotor Mechanisms in Human Communication. Oxford: Oxford University Press.

[B50] Kingstone A. Klein R. (1993). Visual offset facilitates saccadic latency: does pre-disengagement of visuo-spatial attention mediate this gap effect? J. Exp. Psychol. Hum. Percept. Perform. 19, 1251–1265. 8294890 10.1037//0096-1523.19.6.1251

[B51] Kirk R. E. (1982). Experimental Design: Procedures for the Behavioral Sciences, 2nd Edn. Belmont, CA: Cole Publishing Co.

[B52] Kline R. B. (2005). Beyond Significance Testing: Reforming Data Analysis Methods in Behavioral Research. Washington, DC: American Psychological Association.

[B53] Kosslyn S. M. Intrilligator J. M. (1992). Is cognitive neuropsychology plausible? The perils of sitting on a one-legged stool. J. Cogn. Neurosci. 4, 96–106. 23967860 10.1162/jocn.1992.4.1.96

[B54] Lünenburger L. Kutz D. F. Hoffmann K. P. (2000). Influence of arm movements on saccades in humans. Eur. J. Neurosci. 12, 4107–4116. 10.1046/j.1460-9568.2000.00298.x 11069607

[B55] Marrocco R. T. Witte E. A. Davidson M. C. (1994). Arousal systems. Curr. Opin. Neurobiol. 4, 166–170. 7913640 10.1016/0959-4388(94)90067-1

[B56] Mattingley J. B. Berberovic N. Corben L. Slavin M. J. Nicholls M. E. Bradshaw J. L. (2004). The greyscales task: a perceptual measure of attentional bias following unilateral hemispheric damage. Neuropsychologia 42, 387–394. 10.1016/j.neuropsychologia.2003.07.007 14670577

[B57] Maxwell S. E. Kelley K. Rausch J. R. (2008). Sample size planning for statistical power and accuracy in parameter estimation. Annu. Rev. Psychol. 59, 537–563. 10.1146/annurev.psych.59.103006.093735 17937603

[B58] Meyer D. E. Abrams R. A. Kornblum S. Wright C. E. Smith J. E. K. (1988). Optimality in human motor performance: ideal control of rapid aimed movements. Psychol. Rev. 95, 340–370. 3406245 10.1037/0033-295x.95.3.340

[B59] Michael G. A. Ojéda N. (2005). Visual field asymmetries in selective attention: evidence from a modified search paradigm. Neurosci. Lett. 388, 65–70. 10.1016/j.neulet.2005.06.027 16026928

[B60] Mieschke P. E. Elliott D. Helsen W. F. Carson R. G. Coull J. A. (2001). Manual asymmetries in the preparation and control of goal-directed movements. Brain Cogn. 45, 129–140. 10.1006/brcg.2000.1262 11161367

[B61] Miller J. M. (1996). Egocentric localization of a perisaccadic flash by manual pointing. Vision Res. 36, 837–851. 8736219 10.1016/0042-6989(95)00184-0

[B62] Milner A. D. Harvey M. Roberts R. C. Forster S. V. (1993). Line bisection errors in visual neglect: misguided action or size distortion? Neuropsychologia 31, 39–49. 8437681 10.1016/0028-3932(93)90079-f

[B63] Morris S. B. DeShon R. P. (2002). Combining effect size estimates in meta-analysis with repeated measures and independent-groups designs. Psychol. Methods 7, 105–125. 10.1037/1082-989X.7.1.105 11928886

[B64] Oldfield R. C. (1971). The assessment and analysis of handedness: the Edinburgh inventory. Neuropsychologia 9, 97–114. 5146491 10.1016/0028-3932(71)90067-4

[B65] Paillard J. (1982a). Apraxia and the neurophysiology of motor control. Trans. R. Soc. Lond. B Biol. Sci. 298, 111–134. 6125965 10.1098/rstb.1982.0076

[B66] Paillard J. (1982b). The contribution of peripheral and central vision to visually guided reaching, in Analysis of Visual Behaviour, eds Ingle D. J. Goodale M. A. Mansfield R. W. J. (Cambridge: MIT Press), 367–409.

[B67] Palmer T. Tzeng O. J. (1990). Cerebral asymmetry in visual attention. Brain Cogn. 13, 46–58. 2346639 10.1016/0278-2626(90)90039-q

[B68] Perry R. J. Rosen H. R. Kramer J. H. Beer J. S. Levenson R. L. Miller B. L. (2001). Hemispheric dominance for emotions, empathy and social behaviour: evidence from right and left handers with frontotemporal dementia. Neurocase 7, 145–160. 10.1093/neucas/7.2.145 11320162

[B69] Petersen S. E. Posner M. I. (2012). The attention system of the human brain: 20 years after. Annu. Rev. Neurosci. 35, 73–89. 10.1146/annurev-neuro-062111-150525 22524787 PMC3413263

[B70] Poizner H. Klima E. Bellugi U. (1990). What the Hands Reveal about the Brain. Boston, MA: MIT Press.

[B71] Posner M. I. Peterson S. E. (1990). The attention system of the human brain. Annu. Rev. Neurosci. 13, 25–42. 2183676 10.1146/annurev.ne.13.030190.000325

[B72] Poynter W. Roberts C. (2012). Hemispheric asymmetries in visual search. Laterality 17, 711–726. 10.1080/1357650X.2011.626558 23098199

[B73] Pratt J. Bekkering H. Leung M. (2000). Estimating the components of the gap effect. Exp. Brain Res. 130, 258–263. 10.1007/s002219900243 10672480

[B74] Rasmussen T. Milner B. (1977). The role of early left−brain injury in determining lateralization of cerebral speech functions. Ann. N. Y. Acad. Sci. 299, 355–369. 101116 10.1111/j.1749-6632.1977.tb41921.x

[B75] Reuter-Lorenz P. A. Hughes H. C. Fendrich R. (1991). The reduction of saccadic latency by prior offset of the fixation point: an analysis of the gap effect. Percept. Psychophys. 49, 167–175. 2017353 10.3758/bf03205036

[B76] Robertson I. H. Mattingley J. M. Rorden C. Driver J. (1998). Phasic alerting of right hemisphere neglect patients overcomes their spatial deficit in visual awareness. Nature 395, 169–172. 9744274 10.1038/25993

[B77] Rolfs M. Vitu F. (2007). On the limited role of target onset in the gap task: support for the motor-preparation hypothesis. J. Vision 7, 1–20. 10.1167/7.10.7 17997676

[B78] Rothi L. J. G. Heilman K. M. (1997). Apraxia: The Neuropsychology of Action. Hove, UK: Psychology Press.

[B79] Roy E. A. Elliott D. (1989). Manual asymmetries in aimed movements. Q. J. Exp. Psychol. 41, 501–516.

[B80] Roy E. A. Kalbfleisch L. Elliott D. (1994). Kinematic analyses of manual asymmetries in visual aiming movements. Brain Cogn. 24, 289–295. 8185899 10.1006/brcg.1994.1017

[B81] Rushworth M. F. S. Ellison A. Walsh V. (2001). Complementary localization and lateralization of orienting and motor attention. Nat. Neurosci. 4, 656–661. 10.1038/88492 11369949

[B82] Saslow M. G. (1967). Effects of components of displacement-step stimuli upon latency for saccadic eye movement. JOSA 57, 1024–1029. 6035296 10.1364/josa.57.001024

[B83] Schenkenberg T. Bradford D. C. Ajax E. T. (1980). Line bisection and unilateral visual neglect in patients with neurologic impairment. Neurology 30, 509–517. 7189256 10.1212/wnl.30.5.509

[B84] Shallice T. Cooper R. P. (2011). The Organization of Mind. Oxford: Oxford University Press.

[B85] Shulman G. L. Pope D. L. Astafiev S. V. McAvoy M. P. Snyder A. Z. Corbetta M. (2010). Right hemisphere dominance during spatial selective attention and target detection occurs outside the dorsal frontoparietal network. J. Neurosci. 30, 3640–3651. 10.1523/JNEUROSCI.4085-09.2010 20219998 PMC2872555

[B86] Steenhuis R. E. Bryden M. P. (1989). Different dimensions of hand preference that relate to skilled and unskilled activities. Cortex 25, 289–304. 2758854 10.1016/s0010-9452(89)80044-9

[B87] Tabachnick B. G. Fidell L. S. (1989). Using Multivariate Statistics. New York, NY: Harper-Collins.

[B88] Voyer D. Voyer S. D. Tramonte L. (2012). Free-viewing laterality tasks: a multilevel meta-analysis. Neuropsychology 26, 551–567. 10.1037/a0028631 22731609

[B89] Watson N. V. Kimura D. (1989). Right-hand superiority for throwing but not intercepting. Neuropsychologia 27, 1399–1414. 2615939 10.1016/0028-3932(89)90133-4

[B90] Wenban-Smith M. G. Findlay J. M. (1991). Express saccades: is there a separate population in humans? Exp. Brain Res. 87, 218–222. 1756828 10.1007/BF00228523

