# Supplementary material for: Are there right hemisphere contributions to visually-guided movement? Manipulating left hand reaction time advantages in dextrals
Source: Front Psychol. 2015 Aug 28;6:1203. doi: 10.3389/fpsyg.2015.01203 (PMC4551826; doi:10.3389/fpsyg.2015.01203)
Supplement: Supplementary file 1 [file Presentation1.PDF]

CAREY et al. Are there right-hemisphere contributions to visually-guided movement? Manipulating left hand reaction time advantages in dextrals

Other dependent measures Experiment 1. Pointing versus Bisecting

Duration and peak velocity

Movement durations and peak velocities quite consistently favour the dominant hand of right handers in published studies of visually-guided aiming. For duration in hand visible conditions in the present experiment, typical Hand x Hemispace interaction was found ($F_{(1,13)}$=50.12, $p<0.001$), reflecting the usual ipsilateral hemispatial advantages (18 ms for the right hand; 25 ms for the left) that we have described previously in several studies. In hand invisible conditions, the right hand was significantly faster than the left ($F_{(1,13)}$=6.67, $p<0.03$). Even larger Hand x Hemispace effects were obtained ($F_{(1,13)}$=27.69, $p<0.001$; right hand 43 ms; left hand 70 ms) in hand-invisible reaching.

Mean peak velocity was significantly higher in bisecting (by 44 mm/s; $F_{(1,13)}$=5.13, $p<0.05$) in hand visible conditions, and a hand x hemispace interaction of the typical sort was also seen ($F_{(1,13)}$=50.64, $p<0.001$; right hand 92 mm/s faster ipsilaterally; left hand 145 mm/s faster ipsilaterally). In hand-invisible conditions, a comparable Hand x Hemispace effect was obtained ($F_{(1,13)}$=12.74; right hand 80 mm/s faster ipsilaterally; left hand 190 mm/s faster ipsilaterally).

Accuracy

Neither accuracy measure suggested that the absence of hand differences in RT was a consequence of any sort of speed-accuracy trade off. AE and VE were analysed using the same three factor ANOVAS (Task, Hand, Hemispace). In hand-

visible conditions the right hand had lower AE than the left ($F_{(1,13)}$=27.46, p<0.001) and AE was higher for bisecting relative to pointing ($F_{(1,13)}$=30.17, p<0.001). Simple main effects analysis of the task by hand interaction ($F_{(1.13)}$=23.58, p<0.001) suggested that right hand performance was worsened less in bisecting ($F_{(1,13)}$=4.41, p=0.056) than the left hand by removing vision of the hand ($F_{(1,13)}$=5.38, p<0.001).

In hand-invisible conditions, significant Task x Hemispace (F(1,13)=8.66, p<0.02) and Hand x Hemispace (F(1,13)=11.19, p<0.006) interactions were found. Simple main effects of the latter suggests that Hemispace did not affect right hand performance ($F_{(1,13)}$=.264, NS) but that the left hand was, unusually, significantly worse in ipsilateral space (Mean 4.21 cm vs 2.71 cm; $F_{(1,13)}$=7.96, p<0.02).

In terms of our measure of endpoint variability, in hand visible bisection performance was more variable than pointing ($F_{(1,13)}$=27.1, p<0.001). No main effects or interactions were obtained for hand invisible pointing and bisecting.

Other dependent measures Experiment 2.

Duration and peak velocity

For duration in experiment, there was no main effect of Hand ($F_{(1,20)}$=0.3, N.S.), and the usual Hemispace main effect ($F_{(1,20)}$=136.112, p<0.0001) refecting the usual ipsilateral hemispatial advantages (39 ms for the right hand; 45 ms for the left) as described previously and was found in Experiment 1. All higher order interactions were not significant. Peak velocity followed this pattern with significant effects of hemispace ($F_{(1,20)}$=248.3, p<0.0001) but not Hand ($F_{(1,13)}$=0.11, NS) or any higher order interactions. Number of targets did not significantly affect duration or peak velocity.

Other dependent measures Experiment 3. Omnibus analysis only.

Durations and peak velocities

For durations, there were main effects of Task (Gap, No Gap), Hand and Hemispace. The task effect (F(1,20)=6.38, p<0.02) suggests a small speed advantage for no gap conditions of 4 ms. The Hand effect ($F(_{1,20})$=17.84, p<0.001) suggests a small advantage for the right hand of 12 ms. The Hemispace effect ($F(_{1,20})$=264.6, p<0.001) suggests the usually isplateral movement advantages (in this case of 48 ms). Peak velocity was significantly affected by Hand ($F(_{1,20})$=9.77, p<0.004; right hand faster by 81 mm/sec), and Hemispace ($F(_{1,20})$=196.55, p<0.0001; ipsilateral movements were faster by 428 mm/sec).
