# Supplementary material for: Are there right hemisphere contributions to visually-guided movement? Manipulating left hand reaction time advantages in dextrals
Source: Front Psychol. 2015 Aug 28;6:1203. doi: 10.3389/fpsyg.2015.01203 (PMC4551826; doi:10.3389/fpsyg.2015.01203)
Supplement: Supplementary file 2 [file DataSheet1.DOCX]

**An alternative to traditional null hypothesis significance testing estimates of required power**

**MOE=margin of error, which equals 1 arm of a 95% confidence interval.**

**How to estimate required sample size for a precise estimate of the left hand RT effect, according to Cumming (2012):** How large of a confidence interval would still be useful in the context of a given experiment?

In the context of this paper (Carey et al. Are there right hemisphere contributions to visually-guided movement?) If you want to be confident of a reasonably robust left hand RT advantage in any control condition, it makes sense to aim for obtained 95% confidence intervals that would not overlap with zero. From the 5 experiments of the current paper, plus an additional 8 unpublished experiments from our lab (total n=316 right handed participants) we will assume a population mean difference favouring the left hand of µ=7 msec, and a population standard deviation of σ=21 msec. The same studies were used to estimate a population correlation coefficient between right and left hand RT, ρ=0.84.

The Exploratory Software for Confidence Intervals (<http://www.latrobe.edu.au/psy/research/cognitive-and-developmental-psychology/esci>) uses an iterative approach to solve this equation:

N=2(1- ρ)[t_c/100_(N-1)/ƭ]

Where t_c/100_ is the critical value of t for the C% confidence interval, and ƭ represents the fraction of the population standard deviation that an experiment chooses as a target.

The following table provides the ESCI derived estimated for ƭ=0.2 to 0.5. We used a noncentral t distribution rather than z, which is why an iterative approach to these estimates is required; see Cumming & Finch (2001) and Cumming (2012) pp.358-372. We set the critical value of noncentral t for 95% Cis.

| Target MOE SD units | Target MOE in ms | N using noncentral t | N with assurance |
| --- | --- | --- | --- |
| 0.5 | 11.5 | 7 | 12 |
| 0.4 | 8.5 | 11 | 19 |
| 0.3 | 6.5 | 17 | 28 |
| 0.2 | 4.3 | 32 | 50 |

Graphically…..on average, we would expect an MOE of this size….


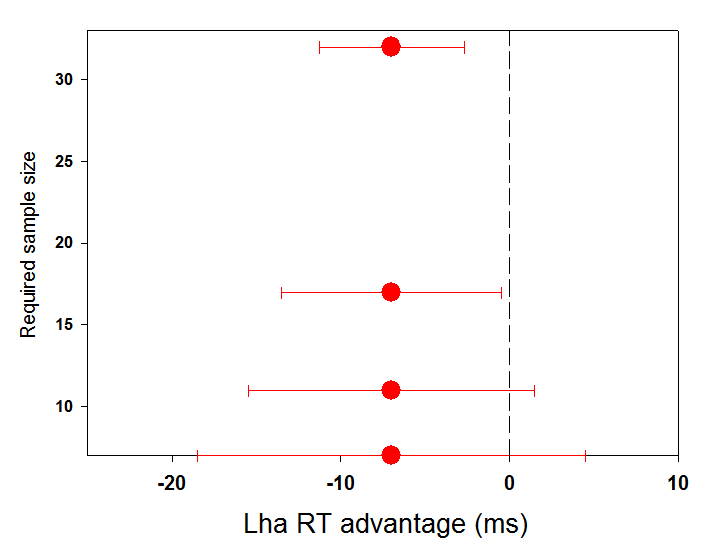


Cumming (2012) also provides advice on an assurance adjustment to these estimates, if scientists want to be sure that 99% of the time we will not exceed the target MOE. These more conservative sample size requirement estimates are plotted below:


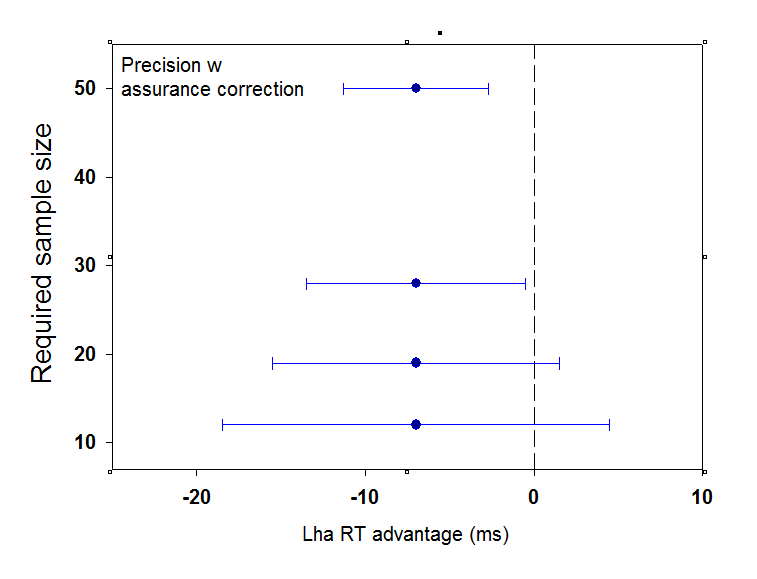


References:

Cumming, G. (2012). *The new statistics: fffect sizes, confidence intervals, and meta-analysis*. Taylor and Francis.

Cumming, G., & Finch, S. (2001). A primer on the understanding, use, and calculation of confidence intervals that are based on central and noncentral distributions. *Educational and Psychological Measurement*, 61(4), 532-574.
